# Prediagnostic Electrocardiographic Abnormalities in Transthyretin Amyloid Cardiomyopathy: A Longitudinal Observational Study

**DOI:** 10.3390/jcm15062201

**Published:** 2026-03-13

**Authors:** Ashwin Venkateshvaran, Helin Mert Karaoglu, Björn Pilebro

**Affiliations:** 1 Institution for Diagnostics and Intervention, Umeå University, 901 87 Umeå, Sweden; 2 Department of Public Health and Clinical Medicine, Heart Centre, 901 87 Umeå, Sweden; helin.karaoglu@me.com (H.M.K.); bjorn.pilebro@umu.se (B.P.)

**Keywords:** transthyretin amyloidosis, ATTR-CM, DPD scintigraphy, heart failure, echocardiography, global longitudinal strain, early diagnosis, wall thickness, SaVR

## Abstract

**Background:** Early diagnosis of transthyretin amyloid cardiomyopathy (ATTR-CM) remains challenging. Although ECG and morphological abnormalities at diagnosis are well-described, their temporal evolution has not been systematically evaluated. This study characterized the prevalence and longitudinal progression of electrical and structural cardiac abnormalities preceding ATTR-CM diagnosis. **Methods:** We retrospectively analyzed patients with confirmed ATTR-CM evaluated at a specialist amyloidosis center between 2006 and 2023. Diagnosis was established by grade 2–3 myocardial uptake on 99mTc-DPD scintigraphy. Standard 12-lead ECGs and transthoracic echocardiograms were reviewed at diagnosis and at baseline, 3–5 years earlier. **Results:** Sixty-three patients (79% men; mean age 77 ± 8 years) were studied, including 33 (52%) with hereditary ATTR (ATTRv) and 30 (48%) with wild-type ATTR (ATTRwt). Overall, 95% had a NAC score ≤ 2, consistent with less advanced disease at diagnosis. During the prediagnostic phase, 79% of patients exhibited pathological ECGs. Non-specific ST–T abnormalities (40%), prolonged QTc (38%), left-axis deviation (35%), first-degree AV block (33%) and anterior infarction pattern (33%) were each observed in at least one-third of patients. From baseline to diagnosis, significant prolongation was observed in the PR interval (+26 ms), QRS duration (+11 ms), and QTc interval (+22 ms) (*p* < 0.001 for all), and a leftward shift observed in the electrical axis (−12.03°, *p* = 0.011). Low voltage was uncommon at both time points. Although interventricular septal thickness increased significantly (+3.42 mm; *p* < 0.001), left ventricular ejection fraction and dimensions were relatively stable. **Conclusions:** In this proof-of-concept study, electrical remodeling precedes functional changes and outperforms low voltages to raise clinical suspicion of ATTR-CM.

## 1. Introduction

Transthyretin amyloidosis (ATTR) is a systemic disorder caused by misfolding of the liver-derived transport protein transthyretin (TTR), leading to extracellular deposition of amyloid fibrils and progressive multiorgan dysfunction. ATTR occurs either as a hereditary disease (ATTRv), caused by pathogenic TTR variants such as the prevalent Val30Met mutation, or as wild-type ATTR (ATTRwt), an age-related systemic disorder. In recent years, ATTR has gained increasing clinical relevance owing to major advances in non-invasive diagnostic techniques and the availability of disease-modifying therapies, which have substantially increased disease awareness and detection [1,2].

Although Val30Met ATTRv often presents with polyneuropathy, cardiac involvement occurs in up to 40% of cases and is the principal determinant of survival [3]. ATTRwt, by contrast, frequently manifests as a cardiomyopathy in older adults and is an increasingly recognized cause of heart failure with preserved ejection fraction. Despite the high diagnostic accuracy of bone-avid tracer scintigraphy [4], most ATTR-CM patients are identified only once cardiomyopathy is advanced and disease-modifying therapy is often initiated late. This diagnostic delay remains clinically relevant, as growing evidence suggests that earlier treatment initiation may slow disease progression and improve outcomes [5,6,7].

Electrocardiographic (ECG) abnormalities such as low QRS voltages, conduction disease, and non-specific repolarization changes are well-recognized ‘red flags’ at the time of diagnosis [3,8]. However, their findings lack sensitivity early in the disease course, and data describing their temporal evolution prior to diagnosis are limited. This represents an important knowledge gap, particularly because infiltration of the conduction system and diffuse interstitial expansion may lead to electrical disturbances before overt echocardiographic abnormalities emerge.

This study aimed to characterize the longitudinal evolution of ECG and echocardiographic abnormalities during the 3–5 years preceding ATTR-CM diagnosis, and to quantify the prevalence of early electrical disease. Specifically, we sought to (1) determine the extent to which conduction disease, repolarization abnormalities, and QTc prolongation are detectable 3–5 years before diagnosis (baseline); (2) evaluate whether early electrical abnormalities differ by ATTR-CM phenotype and (3) assess electrical and structural progression between baseline and diagnosis.

## 2. Methods

**Study Population.** This retrospective study of patients with ATTR-CM prospectively included while evaluated between 2006 and 2023 at Umeå University Hospital, a tertiary-care center of excellence for amyloidosis in Sweden. Diagnosis was established non-invasively using 99mTc-DPD scintigraphy according to the algorithm proposed by Gilmore et al. [4] or through non-cardiac biopsy findings of ATTR deposits in combination with a grade 2+ 99mTc-DPD uptake in the case of ATTRv amyloidosis [4,9]. All patients underwent TTR gene sequencing and prognosticated using NT-proBNP and eGFR (NAC-stage) [10]. Patients with ECGs recorded locally between three and five years prior to diagnosis were included in the study. The study was conducted according to the guidelines of the Declaration of Helsinki, and approved by the Ethics Committee in Umeå (protocol codes Dnr 06-084 approved 7th June 2006 and Dnr: 2016-435-31M approved 7th February 2017.)

**Electrocardiography.** All electrocardiograms analyzed in this study were standard 12-lead ECG examinations (50 mm/s, 10 mm/mV) collected from the patients’ clinical records. Each ECG was analyzed for rhythm, frontal plane/electrical axis and QRS amplitudes, as well as for the presence of ventricular pacing, conduction abnormalities, ST- and T abnormalities, and anterior infarction pattern. The interpretation of the electrocardiographic examinations was conducted in accordance with the recommendations of the American Heart Association, The American College of Cardiology foundation and the Heart Rhythm Society [11,12,13,14]. An exception concerned the classification of left anterior hemiblock (LAH). In order to capture early fascicular conduction abnormalities potentially related to amyloid infiltration, LAH was defined by the presence of marked left-axis deviation (≤−45°) in the absence of left bundle branch block or ventricular pacing, irrespective of QRS duration. Accordingly, patients with concomitant right bundle branch block or non-specific intraventricular conduction delay were classified as having LAH when these criteria were met. This definition was chosen to reflect left anterior fascicular involvement rather than guideline-defined fascicular block. All amplitudes were measured manually and are presented in millivolts (mV). Low voltage was defined as a maximal amplitude of <0.5 mV in limb leads, and/or a maximal amplitude of <1.0 mV in precordial leads. QT intervals were obtained from automated measurements for all ECGs and were not reported for patients with bundle branch block or ventricular pacing. QTc was calculated using Bazett’s formula. In patients with atrial fibrillation or flutter, automated QT measurements were visually verified by manual measurement using three consecutive beats. These manual measurements showed minimal differences, and therefore the automated measurements were retained for analysis. Anterior infarction pattern was defined using Minnesota code criteria I.1, I.2 or I.3 [15]. For the purposes of this study, some ECG abnormalities were not included in the analysis as they are hard to clearly define (interventricular conduction defects and incomplete bundle branch blocks) or situational (sinus tachycardia, sinus bradycardia and status post inferior infarction).

**Echocardiography.** Comprehensive transthoracic echocardiography was performed by experienced echocardiographers using Vivid 7, E9 or E95 ultrasound systems (GE Ultrasound, Horten, Norway) according to current international recommendations [16]. Digital loops were stored, exported and analyzed offline (EchoPac v204, GE Ultrasound, Waukesha, Wisconsin). Standard 2D measurements in the parasternal long-axis (PLAX) view included left ventricular end-diastolic diameter (LVDD), interventricular septal thickness in diastole (IVSD) and posterior wall thickness (PWT). Relative wall thickness (RWT) was assessed as 2× (PWT/LVDD). Ejection fraction (EF) was measured by Simpsons biplane method.

**Statistical analysis.** Continuous variables were assessed for normality using Q–Q plots and the Shapiro–Wilk test. Normally distributed variables were compared using paired *t*-tests, while non-normally distributed variables were assessed using Wilcoxon signed-rank tests. McNemar’s test was used to compare paired categorical variables. Wald intervals were reported for all proportions. A two-sided *p*-value < 0.05 was considered statistically significant. Longitudinal changes in continuous ECG, echocardiographic, and biomarker variables were assessed using linear mixed-effects models with time point as a fixed effect and patient ID as a random intercept to account for within-subject correlation. Analyses were performed using IBM SPSS version 28 for Windows (IBM Corp., Armonk, NY, USA).

## 3. Results

**Figure 1:**
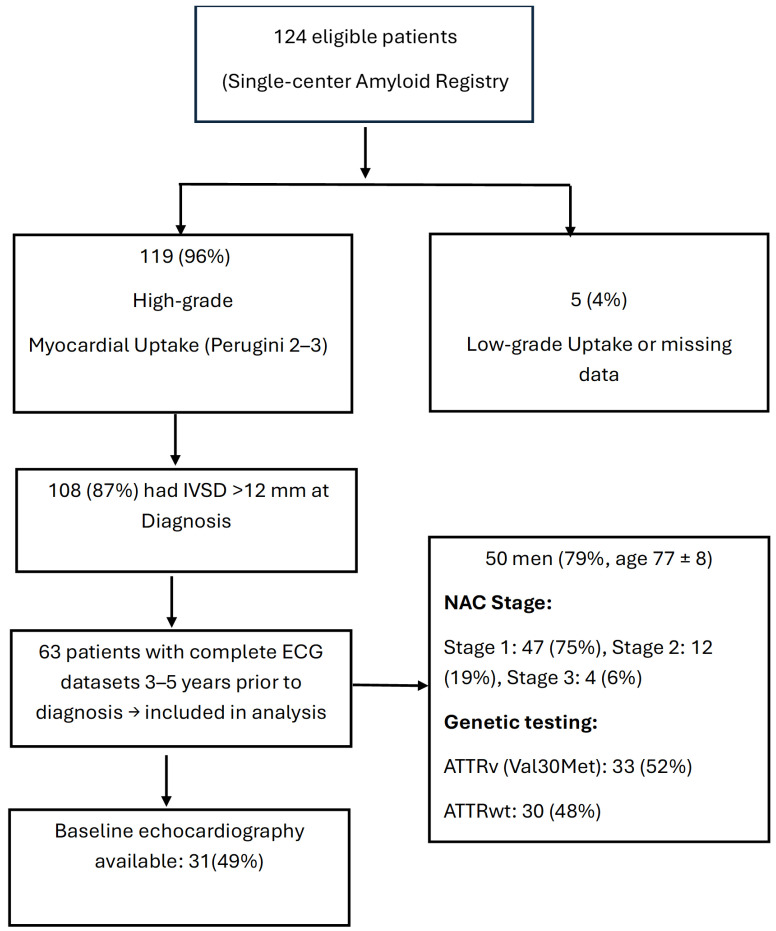
Patient selection flow chart for the study cohort.

**Study Population.** Among 124 eligible patients in this single-center amyloid registry, 119 (96%) had high-grade myocardial uptake (Perugini score 2–3). Intraventricular septal diameter >12 mm at diagnosis was observed in 108 (87%) patients. A total of 63 patients had complete ECG datasets recorded between 3 and 5 years prior to diagnosis and were included in the analysis (50 men; age 77 ± 8 years). The majority of the patients demonstrated a low NAC stage, indicating that diagnosis of cardiac amyloidosis was performed reasonably early: 47 of 63 (75%) had a score of 1, 12 (19%) a score of 2, and only 4 (6%) a score of 3 [10]. Genetic testing revealed Val30Met variant in 33 patients (52%; age 73 ± 7 years). The remaining patients had normal genetic workup (48%; age 83 ± 5 years) (Figure 1).

**Electrical and Structural Variables at Diagnosis.** ECG and echocardiographic data are presented in Table 1. Electrocardiographic data were complete at diagnosis and baseline. Echocardiographic data at diagnosis was available for all patients; baseline data was available in 31 patients (49%). No differences were observed in age, sex distribution or ATTR subtype at diagnosis between patients with and without baseline echocardiographic data. At diagnosis, 97% of patients had at least one of the defined ECG abnormalities, with more than half of the cohort demonstrating first-degree AV block, left-axis deviation, anterior infarction pattern, ST–T abnormalities, or prolonged QTc. When considering structural variables, IVSD, PWT, and RWT were elevated above normal reference ranges. The majority of patients (N = 46; 73%) displayed EF ≥ 50%, 7 (11%) displayed mildly reduced EF between 40 and 49%, and 10 (16%) displayed EF < 40%.

**Figure 2:**
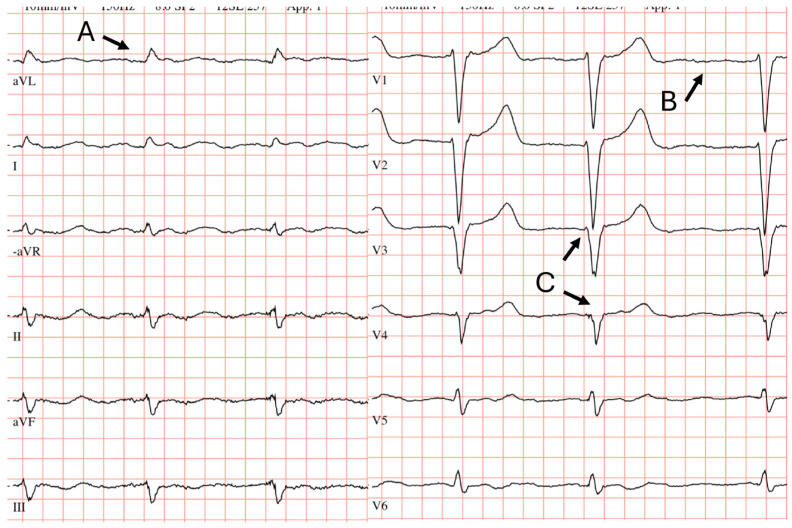
Twelve-lead ECG demonstrating left-axis deviation (A), atrial fibrillation (B), and anterior infarction pattern in the precordial leads (C) seen in an ATTR-CM patient 3–5 years before diagnosis.

**Electrical and Structural Variables at Baseline.** Even 3–5 years prior to diagnosis, electrical abnormalities were observed in 79% of patients. An illustration of an ECG taken at this time is provided in Figure 2. Non-specific ST–T abnormalities (40%), prolonged QTc (38%), left-axis deviation (35%), first-degree AV block (33%) and anterior infarction pattern (33%) were observed in at least one in three patients (Table 1). When AV block, left-axis deviation and QTc prolongation were considered, 32% of the cohort exhibited at least two of these abnormalities. Interestingly, low voltage was uncommon both at baseline and diagnosis (3% and 5% respectively). Structural abnormalities were present at baseline, with 42% of patients displaying IVSD > 14 mm, 29% with PWT > 10 mm, and 42% with RWT > 0.42. Nearly one-third (29%) exhibited severe septal hypertrophy (IVSD ≥ 16 mm). Among patients with IVS thickness ≤ 14 mm (n = 19/31), a substantial proportion already exhibited electrocardiographic abnormalities. Specifically, four patients (21%) had first-degree AV block, four patients (21%) had left anterior hemiblock (LAH), five (26%) had anterior infarction pattern and five patients (26%) had QTc prolongation.

**Subgroup Analyses.** At diagnosis, ATTRwt displayed higher heart rate (HR) (81 ± 16 vs. 71 ± 10 bpm, *p* = 0.01), lower SV (61 ± 12 vs. 81 ± 20 mL, *p* < 0.001) and CO (4.3 ± 0.9 vs. 5.6 ± 1.5 L/min; *p* = 0.02) and higher mitral E/A ratio (2.0 ± 0.9 vs. 1.2 ± 0.6; *p* = 0.02) compared with ATTRv. Electrical and echocardiographic variables at baseline between ATTRwt and ATTRv are presented in Table 2. ATTRwt showed a higher prevalence of atrial fibrillation (AF), lower prevalence of LAH and anterior infarction pattern than ATTRv (*p* < 0.05 for all comparisons). R-wave amplitude in aVR (R-aVR) was higher in ATTRwt compared with ATTRv. Echocardiographic variables did not differ between groups.

**Progression of Electrical and Structural Variables.** To evaluate the temporal evolution of cardiac involvement during the 3–5 years preceding diagnosis, linear mixed-effects models were employed to account for within-subject correlation. The median interval baseline-to-diagnosis interval was 49 months (IQR 26–69) for ECG and 50 months (IQR 29–64) for echocardiography. From baseline to diagnosis, there were marked increases in the PR interval (Estimate: +25.98 ms, *p* < 0.001), QRS duration (Estimate: +11.08 ms, *p* < 0.001), and QTc interval (Estimate: +21.79 ms, *p* < 0.001). Furthermore, a significant leftward shift in the electrical axis was observed (Estimate: −12.03°, *p* = 0.01). Interestingly, QRS amplitudes remained largely unchanged over the study period (*p* > 0.05), and low voltage remained uncommon at both time points. Concomitant with greater amyloid burden, IVSD increased significantly by an average of 3.42 mm (*p* < 0.001). However, both EF (Estimate: −1.5, *p* = 0.29) and LVDD (Estimate: −0.91) remained relatively stable. NT-proBNP level nearly tripled from baseline to diagnosis (*p* < 0.001) (Table 3).

## 4. Discussion

In this proof-of-concept, single-center, observational study, we highlight the value of routine electrocardiography for diagnostic use even in early stages of ATTR-CM. We found that 79% of patients already exhibit pathological ECG findings during the prediagnostic stage, with left-axis deviation, progressive conduction delay, and QTc prolongation emerging as early electrical signals that may prompt suspicion for ATTR-CM. In addition, longitudinal progression was characterized by worsening electrical abnormalities concomitant to rising cardiac biomarkers and increasing myocardial wall thickness, while left ventricular size and systolic function remained relatively preserved. Taken together, these findings are hypothesis-generating and suggest a potential role for combinations of ECG variables as accessible, routinely available screening tools during the early phase of ATTR-CM.

**Pathophysiological Interpretation of Early Electrical Remodeling.** Our observation that 79% of patients had pathological ECG findings 3–5 years before diagnosis indicates that electrical remodeling is an early hallmark of ATTR-CM. This likely reflects amyloid deposition in two key anatomical compartments. First, involvement of the His–Purkinje system, evidenced by prolongation of the PR interval and QRS duration, consistent with the high affinity of amyloid for specialized conduction tissue. Deposition often begins in the sub-endocardium, directly impairing rapid conduction pathways before substantial transmural structural remodeling occurs [17]. Second, interstitial myocardial deposition may account for the QTc prolongation. Amyloid infiltration in this compartment may physically separate myocytes and alter the distribution or function of ion channels, promoting heterogeneous ventricular repolarization [18]. These findings suggest that electrical remodeling precedes overt structural changes and may offer a window for earlier detection and risk stratification in ATTR-CM.

**Low QRS voltages as Diagnostic pitfalls in ATTR-CM.** Our study highlights a critical diagnostic pitfall concerning over-reliance on low QRS voltage as a “red flag” for amyloidosis. Unlike AL amyloidosis, where low voltage is common due to light-chain-mediated myocyte toxicity, low voltage was rare in our ATTR-CM cohort (3% at baseline, 5% at diagnosis) despite a significant increase in septal thickness. Observing reductions in QRS amplitudes during the prediagnostic period, these observations suggest that low voltage is probably a sign of very advanced amyloid infiltration in ATTR-CM. This also suggests that the substantial mass of amyloid fibrils and associated myocyte hypertrophy in ATTR may contribute to the surface electrical signal and mask the classic low voltage phenotype historically associated with cardiac amyloidosis [19]. Earlier studies have documented high QRS voltages in hypertensive LVH or aortic stenosis with increased wall thickness, providing subtle but valuable clues that may help distinguish ATTR-CM from these common mimics [20]. Importantly, ECG evaluation that considers QRS voltage in relation to left ventricular mass or interventricular septal thickness—even when voltages are not formally “low”—remains a useful method to raise suspicion for ATTR-CM [21]

**Comparison between ATTR subgroups.** The comparison between ATTRv and ATTRwt is exploratory and should be interpreted cautiously. ATTRwt patients were approximately 10 years older, and age is a major determinant of atrial fibrillation and several ECG abnormalities. As expected, atrial fibrillation was more prevalent in ATTRwt. Notably, however, aside from atrial fibrillation, several conduction abnormalities were numerically more frequent in the younger ATTRv subgroup despite similar degrees of echocardiographic remodeling. While this observation may suggest earlier or more pronounced conduction system involvement in ATTRv, the small sample size and lack of age-adjusted analyses preclude firm conclusions. These findings should therefore be regarded as hypothesis-generating and warrant confirmation in larger, appropriately adjusted cohorts.

**Clinical Implications.** The progressive nature of these electrocardiographic markers underscores the utility of the ECG as a low-cost, longitudinal screening tool in at-risk populations. We propose further investigations into the concept of an “ECG red-flag cluster,” comprising first-degree AV block, left-axis deviation, and QTc prolongation, to prompt heightened diagnostic vigilance. Importantly, these features are relatively common in older adults and are non-specific; thus, they should not be interpreted in isolation as a trigger for the diagnostic workup of cardiac amyloidosis. Instead, their diagnostic value is maximized when considered in combination with other cardiac and extracardiac red flags, including the mismatch between QRS voltages and left ventricular mass, as well as the overall clinical context. The presence of these patterns in older patients may serve as a valuable prompt for timely evaluation with echocardiography, bone scintigraphy, or genetic testing, even in the absence of overt symptoms or in cases of borderline left ventricular wall thickness. Incorporating such a cluster into routine assessment may facilitate earlier detection of ATTR-CM and enable intervention before advanced structural remodeling occurs. However, the diagnostic utility of these findings needs to be evaluated in studies including control populations with and without other cardiac conditions, and in combination with previously suggested cardiac and extracardiac red flags.

**Limitations.** Our study is limited by its small patient population, single-center retrospective design and the potential for both survivor bias and a bias towards patients with longer disease duration. The decrease in the final analytical cohort from 108 patients with septal hypertrophy to 63 was driven by the strict requirement for high-quality, archival ECG datasets from the specific 3–5-year prediagnostic window. We cannot rule out that the archived ECG recordings were not performed due to early symptoms of ATTR-CM. This bias is probably even stronger for patients who had undergone echocardiography and NT-proBNP testing. Additionally, due to ethical and regulatory restrictions, we were unable to access comprehensive medical records from non-cardiology care providers. As a result, detailed information regarding symptoms or the clinical indications for ECG acquisition at the prediagnostic timepoint was not available, limiting our ability to more precisely define disease stage at baseline. Baseline echocardiographic data were available in only 49% of the cohort, which may impact the comparison of structural progression rates. Additionally, the classification of left anterior hemiblock (LAH) in this study differs from strict guideline definitions, as it was intended to capture early fascicular conduction abnormalities associated with amyloid infiltration rather than describe the isolated finding. While this approach highlights left-axis deviation as an important sign for detecting early electrical disease, it may limit direct comparability with studies adhering strictly to guideline-based criteria. The vast majority of patients were, however, at the time of diagnosis, in NAC-stage 1, indicating that ATTR-CM was not very long progressed at the time of diagnosis. While this ensures the robustness of the longitudinal data, it may limit the generalizability of the findings. Finally, the definition of a pathological ECG was restricted to major and reproducible abnormalities. Several findings with limited consensus definitions or potential situational variability were not classified as pathological, which may have influenced prevalence estimates and limits comparability with studies using broader ECG definitions.

**Conclusions.** In this proof-of-concept study, early ECG abnormalities, including conduction delays, left-axis deviation, and QTc prolongation, were common 3 to 5 years prior to diagnosis and may be more sensitive than low QRS voltages for raising suspicion of ATTR-CM. These hypothesis-generating findings require validation in larger, controlled studies.

## Figures and Tables

**Table 1:** Electrocardiography and echocardiography data of patient cohort at diagnosis and baseline (3–5 years earlier). *p*-value represents differences between values at baseline and diagnosis.

Variables	At Baseline	At Diagnosis	*p*-Value
**ECG**			
Any abnormalities	50/63 (79%)	61/63 (97%)	<0.001
Atrial fibrillation/flutter	11/63 (18%)	20/63 (32%)	0.01
Atrial pacing	0/63 (0%)	3/63 (5%)	0.08
AV-block I	17/52 (33%)	20/40 (50%)	0.01
Ventricular pacing	3/63 (5%)	7/63 (11%)	0.001
Left axis deviation	21/60 (35%)	34/57 (60%)	<0.001
Left anterior hemiblock	14/58 (24%)	19/54 (35%)	0.008
Left bundle branch block	2/60 (3%)	3/57 (5%)	0.15
Right bundle branch block	5/60 (8%)	15/57 (27%)	0.002
Anterior infarction pattern	20/60 (33%)	35/57 (61%)	<0.001
ST-T abnormalities	21/53 (40%)	23/39 (59%)	<0.001
Pathological QTc	23/60 (38%)	36/57 (63%)	0.002
Low voltage	2/60 (3%)	3/57 (5%)	0.65
PR interval (msec)	186 ± 34	209 ± 44	<0.001
QRS duration (msec)	98 ± 19	109 ± 23	<0.001
Electrical axis	−11 ± 44	−22 ± 61	0.012
QTc	444 ± 28	466 ± 31	<0.001
**Echocardiography**			
LVDD (mm)	49 ± 6.3	46 ± 5.5	<0.001
IVST (mm)	14 ± 3.4	17 ± 3.2	<0.001
PWT (mm)	10 ± 1.9	12 ± 2.5	<0.001
RWT	0.43 ± 0.12	0.52 ± 0.13	<0.001

**Abbreviations:** LVDD = left ventricular diastolic diameter, IVST = interventricular septal thickness in diastole; PWT = posterior wall thickness in diastole; RWT = relative wall thickness.

**Table 2:** Electrocardiography and echocardiography data at baseline stratified by wild-type (ATTRwt) or variant (ATTRv) subgroup. *p*-value represents differences between ATTR-CM subgroups.

Variables	ATTRwt (n = 30)	ATTRv (n = 33)	*p*-Value
**ECG**			
Atrial fibrillation/flutter	9/30 (30%)	2/33 (6%)	0.01
Atrial pacing	0/30 (0%)	0/33 (0%)	0.49
AV-block I	9/30 (30%)	10/33 (30%)	0.44
Ventricular pacing	2/30 (7%)	1/33 (3%)	0.25
Left anterior hemiblock	3/28 (11%)	11/30 (37%)	0.02
Left bundle branch block	0/30 (0%)	2/33 (6%)	0.37
Right bundle branch block	2/30 (7%)	3/33 (9%)	0.25
Anterior infarction pattern	5/28 (18%)	15/22 (68%)	0.01
ST-T abnormalities	12/26 (46%)	8/27 (30%)	0.21
Low voltage	0/28 (0%)	2/32 (6%)	0.17
PR interval (msec)	187 ± 29	184 ± 27	0.76
QRS duration (msec)	96 ± 13	100 ± 23	0.45
Electrical axis	−0.75 ± 41	−15 ± 45	0.24
QTc	441 ± 26	447 ± 28	0.31
R-aVR (mV)	5.9 ± 2.5	4.4 ± 2.2	0.008
**Echocardiography**			
LVDD (mm)	51 ± 7	48 ± 6	0.20
IVST (mm)	13.7 ± 3	14 ± 3	0.79
PWT (mm)	11 ± 1.7	11 ± 1.5	0.98
RWT	0.44 ± 0.13	0.46 ± 0.09	0.07

**Abbreviations:** LVDD = left ventricular diastolic diameter, IVST = interventricular septal thickness in diastole; PWT = posterior wall thickness in diastole; RWT = relative wall thickness.

**Table 3:** Longitudinal analysis of cardiac amyloidosis progression: continuous ECG, echocardiographic, and biomarker variables evaluated by linear mixed-effects models.

Variable	Mean (Baseline)	Mean (Diagnosis)	Estimate (Time Effect)	*p*-Value
NT-proBNP †	1053.3	3262.5	+1.190	<0.001
IVSD	13.43	16.73	+3.419	<0.001
PR Interval (ms)	185.5	207.9	+25.98	<0.001
QRS Duration (ms)	97.9	108.6	+11.08	<0.001
QTc (ms)	444.4	465.8	+21.79	<0.001
R-aVR (mV)	0.51	0.37	−0.153	<0.001
Heart Rate	70.4	76.0	+5.619	0.013
Electrical Axis	−11.0	−22.1	−12.03	0.011
RVSP	36.1	39.4	+4.259	0.019
EF	51.4%	50.6%	−1.581	0.292
LVDD	47.16	46.33	−0.913	0.574

**Abbreviations:** ECG = electrocardiogram; EF = ejection fraction; IVSD = interventricular septal thickness in diastole; LVDD = left ventricular end-diastolic diameter; NT-proBNP = N-terminal pro-B-type natriuretic peptide; PR = PR interval; QRS = QRS duration; QTc = corrected QT interval; R-aVR = R-wave amplitude in lead aVR; RVSP = right ventricular systolic pressure. Values are expressed as Mean ± SD unless otherwise noted. Estimates and *p*-values are derived from linear mixed-effects models with Timepoint as a fixed effect and Patient ID as a random intercept. † NT-proBNP values were log-transformed for analysis due to non-normal distribution; the estimate represents the change in log-transformed units.

## Data Availability

The data that support the findings of this study are available from the corresponding author upon reasonable request.
